# A Cancer Gene Module Mining Method Based on Bio-Network of Multi-Omics Gene Groups

**DOI:** 10.3389/fonc.2020.01159

**Published:** 2020-06-19

**Authors:** Chunyu Wang, Ning Zhao, Kai Sun, Ying Zhang

**Affiliations:** ^1^School of Computer Science and Technology, Harbin Institute of Technology, Harbin, China; ^2^School of Life Science and Technology, Harbin Institute of Technology, Harbin, China; ^3^Thoracic Surgery Department, General Hospital of Heilongjiang Province Land Reclamation Bureau, Harbin, China; ^4^Department of Pharmacy, General Hospital of Heilongjiang Province Land Reclamation Bureau, Harbin, China

**Keywords:** multi-omics data, bio-network model, compactness clustering, lncRNA, dysregulation

## Abstract

The initiation, promotion and progression of cancer are highly associated to the environment a human lives in as well as individual genetic factors. In view of the dangers to life and health caused by this abnormally complex systemic disease, many top scientific research institutions around the world have been actively carrying out research in order to discover the pathogenic mechanisms driving cancer occurrence and development. The emergence of high-throughput sequencing technology has greatly advanced oncology research and given rise to the revelation of important oncogenes and the interrelationship among them. Here, we have studied heterogeneous multi-level data within a context of integrated data, and scientifically introduced lncRNA omics data to construct multi-omics bio-network models, allowing the screening of key cancer-related gene groups. We propose a compactness clustering algorithm based on corrected cumulative rank scores, which uses the functional similarity between groups of genes as a distance measure to excavate key gene modules for abnormal regulation contained in gene groups through clustering. We also conducted a survival analysis using our results and found that our model could divide groups of different levels very well. The results also demonstrate that the integration of multi-omics biological data, key gene modules and their dysregulated gene groups can be discovered, which is crucial for cancer research.

## Introduction

Studies in systems biology have revealed that cell functions in biological systems generally involve the interaction of multiple genes (1). In this era of functional genomics, many gene groups have been pinpointed using different high-throughput technologies, such as micro-matrix technology, mass spectrometry analysis technology, and next-generation sequencing technology (2–5). These gene groups are often associated with many similar diseases, and are usually used as novel protein complex, differentially expressed genes or co-expressed gene module, and sometimes in effector signaling pathway in further downstream research (6). It is important, however, to compare gene groups and interpret the correlation between them as potential biological mechanisms that are easy to understand.

In recent years, an emerging number of new computing techniques and utilities have been developed to explore such gene groups. Most of them for analyzing gene functions are enrichment analysis tools. An important part of these bioinformatics enrichment analysis software adopts Gene Ontology (GO) (7), which only allows simple single group analysis and identifies GO terms overrepresented within the group. For example, the tools GOstat (8), GO::TermFinder (9), and GOEAST (10) are often used for GO analysis. To implement a single gene group functional analysis, some of them integrate diversify integrated heterogeneous data, such as Gazer (11), GeneTrail (12), DAVID (13), and GSEA (14). However, even in complete biological pathways, there are no genes that act alone (15). Gene groups collaborate with other ones through sophisticated technique, and these interrelations can affect related disease and phenotype. Therefore, the prevailing challenge binding biologists is to obtain easy-to-understand functional links between different gene groups in the background of this complexity. At present, some calculation tools based on GOs have been developed for comparing gene group relationships, such as FatiGO (16) and ProfCom (17). Some semantic similarity methods based on GO can also compare between gene groups by the mean of the pairwise distances of different elements (18). Essentially, GO acts as only language annotations, and it is still challenging to make GO gene annotations accurately indicate the substantial intricacy of biological functions and relations (19). Others directly compare literature keywords to reflect the links between groups (20). Some calculation tools using these keywords are based on the same idea, and use GO terms or literature citations to map biological knowledge to gene groups, and then find significantly overrepresented GO terms or keywords. This methodology, however, cannot provide a functional similarity measurement between gene groups.

Protein-Protein Interaction Networks (PPINs) can reveal the functional interaction in proteins (21–36). The closeness of proteins within a network is a sign of the similarity of their functions. It also suggests that the diseases caused by the genes represented by these proteins are the same. In terms of predicting carcinogenic gene patterns, some methods use PPINs and disease phenotype similarity data to calculate and rank genes to screen for carcinogenic genes. Oti et al. (37) predicted carcinogenic genes by integrating PPINs and genetic loci. Their work illustrates the importance of PPINs for predicting carcinogenic genes. The drawback of this method is that there is often noise in a PPIN, and the source of some disease sites is not accurate, meaning the derivation process is not perfect. Lage et al. (38) proposed a novel method for calculating the association score between genes and cancer, which was based on the information that candidate carcinogenic genes within one step of a PPIN act on the same or similar diseases, allowing them to calculate the association score, and further mine candidate carcinogenic genes and cancer-associated protein complexes. Goldenberg et al. (39) used high differential expression changes in normal and abnormal cancer data to establish a regression model and identify a set of genes with consistent expression changes of neighboring genes in their network. It was proposed that this set of genes causes changes in the expression of neighboring genes.

In terms of cancer-related disorder modules and pathways, these modules and pathways are often based on a PPIN, studied by collecting differentially expressed genes in normal samples and tumor samples and using them as candidate genes, and then mining the disorder modules through different analysis methods. Chowdhury et al. (40) considered the mutual information between the dominantness of samples and the dominantness of samples with known gene expression, used information theory to establish a subnet state function to find subnetworks with coexpression disorders, and used neural network models as classification detection model in this method. Vandin et al. (41) provided a method (Dendrix) for discovering mutation-driven pathways in somatic mutation data, which was different from the discovery of genes with effective frequency mutations in cancer genomes (42–49), that is, those driver mutations that targeted multiple cell signals and regulatory pathways. Backes et al. (50) proposed a method to identify abnormal regulatory modules and related important genes. This method focused on abnormal regulation subgraphs, and its detection method was based on a new pruning strategy. This method was not only applicable to directed networks, but also applicable to undirected networks. Proteins rarely act alone usually; instead they form networks of complex interactions among different molecules. Biological cell functions are commonly accomplished in a hierarchical manner (51). Related researches have exposed that PPINs can reflect the protein functional interactions. The distance between proteins is highly correlated to their function similarity within a network (52–57).

**Figure 1:**
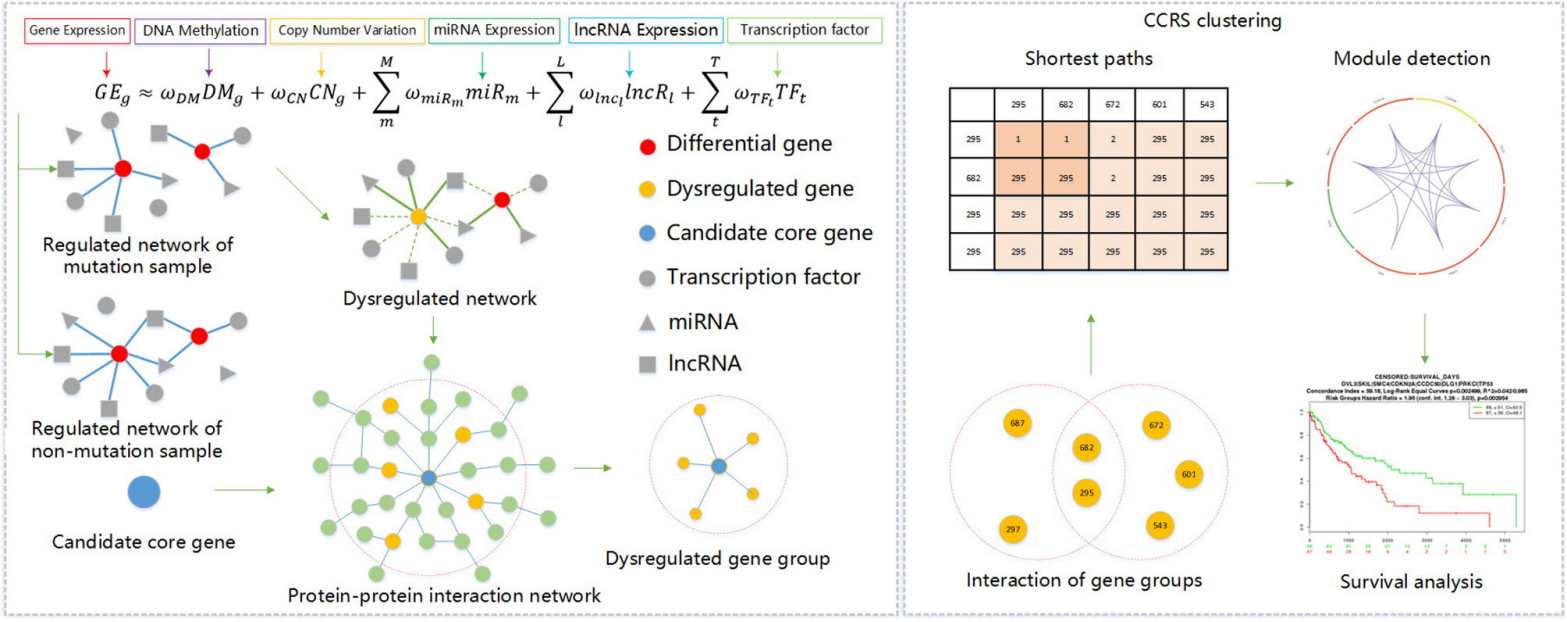
Framework of core module mining method.

Given these challenges, we analyzed the integration mode of data at different levels, and through experimental analysis and verification, we scientifically introduced lncRNA expression profiling data to further expanded the breadth of multi-omics data integration methods. We then mined carcinogenic gene modules composed of candidate genes, and through a corrected cumulative rank score method, we were better able to understand the interaction level of gene groups in a PPIN and calculated the functional similarity score between gene groups. We used the corrected cumulative rank score as distance measure for a compactness clustering method, and mined the gene modules hidden in the key genes of this abnormal regulation gene group. The specific process description of this method is shown in Figure 1.

## Methods

### Multi-Omics Data

We downloaded Lung squamous cell carcinoma (LUSC) multi-omics data from TCGA database, including DNA methylation, copy number variation (CNV), miRNA and gene expression data. We directly derived somatic variation data using 338 samples from the TCGA database. By filtering silent mutations, a set of 5,982 mutant genes was obtained. The CNV data was processed using GISTIC software (58) and CNV matrix data for 337 samples was obtained. We downloaded the DNA methylation data of 367 samples tested using the JHU-USC HumanMethylation450 platform, and obtained the expression data of a total of 20,503 genes from 337 samples, the expression data of 1,043 miRNAs from 387 samples, and downloaded the expression data of 12,728 lncRNAs from 220 samples from the TANRIC database (59). Finally, 66 LUSC samples with all multi-omics data were identified and used for study.

miRNA-target data was obtained from StarBase. To reduce false positives, the following screening criteria were selected: (1) at least one CLIP-Seq test supported the miRNA target site; (2) miRNA and target gene expression were negatively correlated in at least three cancers (Pearson correlation: γ < 0, *p* < 0.05); (3) the target relationship was predicted using at least one of different five prediction software platforms (TargetScan, PicTar, PITA, miRanda/mirSVR, and RNA22). We obtained experimentally verified the regulatory relationships of transcription factor (TF) genes in TRANSFAC database (60) and used conservative TF binding sites in the UCSC genome browser. Finally, from BioGRID, BIND, HPRD, IntAct, MINT, MIPS, PDZBase, DIP, and Reactome databases, we collected human protein-protein interaction data. We also remove all redundant data which have not been experimentally verified or predicted in related literatures.

The relationship between lncRNA and gene function was obtained as follows. We know that each miRNA has a regulatory relationship with genes, and miRNAs also have a regulatory relationship with lncRNAs. Thus, we assumed that genes and lncRNAs regulated by identical miRNAs were also interactive. For genes and lncRNA regulated by the same miRNA, we calculated the Pearson correlation (γ < 0, *p* < 0.05) of the gene expressions from our 66 samples to obtain the links between lncRNAs and genes. Multi-omics biological data is shown in Table 1. The data on the regulatory relationship of regulatory factors to these genes are listed in Table 2, including numbers of regulatory factors and genes.

**Table 1:** Multi-omics biological data.

**Name**	**Number of samples**	**Number of regulatory factors**
Gene expression	367	20,502
miRNA expression	338	223
DNA Methylation	367	19,355
lncRNA expression	66	7,274
DNA copy number	367	23,109

**Table 2:** Regulatory relationship data.

**Name**	**Number of regulatory factors**	**Number of genes**
TF-gene	639	5,970
miRNA-Gene	224	12,323
lncRNA-gene	286	2,705

### Abnormal Regulatory Gene Group

First, we selected genes from DNA mutation data to obtain all the genes that produced mutations, constructed a gene mutation spectrum, and then used GISTIC software to process the DNA CNV data to determine the discretization value of CNV for each gene in all cancer samples (−2, −1, 0, 1, 2) to obtain the DNA CNV spectrum.

With the gene mutation spectrum and CNV spectrum data collectively, a binary gene variation matrix *M* was generated. The matrix rows corresponded to genes, and columns to samples. If the gene of the *i*-th row had both gene mutations and CNVs in the sample of the *j*-th column, *m*_*ij*_ was assigned value 1 though, otherwise assigned 0. The criteria for screening key candidate genes were: (1) the samples should produce more than ten percent of total mutations in the gene variation matrix; and, (2) the gene produced differential expression in the mutated and non-mutated samples. The second screening criterion required the integration of gene expression profiling data. A Student's *T*-test was performed between the mutated and non-mutated samples of the gene, and the standard was that the false discovery rate was <0.01. Through the above two criteria, we obtained mutation information for key candidate genes.

An abnormal regulatory network was constructed for every key candidate gene, and it was then necessary to distinguish between mutated samples and non-mutated samples for every abnormal regulatory network. The cancer samples were divided into mutated and non-mutated samples, and then a linear regression model was constructed to describe the variation in gene expression. The variables in the regression model included the DNA methylation, CNV, TFs that regulate the particular gene, miRNAs, and lncRNAs.

Under the specific conditions of including *G* samples, given a gene *g*, there were *T* TFs(TF_1_, TF_2_, …, TF_*T*_), *M* miRs(miRNA_1_, miRNA_2_, …, miRNA_*M*_) and *L* lncRs(lncRNA_1_, lncRNA_2_, …, lncRNA_*L*_) are bound with *g*. We can then train a linear regression model using the following formula:

(1) $$\begin{array}{l} GE_{g}\approx {\omega_{DM}DM_{g}+\omega }_{CN}CN_{g}+\overset{M}{\underset{m}{\sum }}\omega_{miR_{m}}miR_{m}\\ +\overset{L}{\underset{l}{\sum }}\omega_{\ln\;c_{l}}lncR_{l}+\overset{T}{\underset{t}{\sum }}\omega_{TF_{t}}TF_{t} \end{array}$$

where *GE*_*g*_ is the expression level of *g* in *G* samples, *CN*_*g*_ is the occurrence times of copy number of *g*, *DM*_*g*_ keeps methylation value of *g*, *TF*_*t*_ keeps expression level of the *t*-th TF that regulates *g*, *miR*_*m*_ saves expression level of the *m*-th miRNA targeting *g*, and *lncR*_*l*_ is the expression level of the *t*-th lncRNA that affects *g*. ω_*CN*_, ω_*DM*_, ω_*T*_*F*__*t*__, ω_*mi*_*R*__*m*__, ω_ln_*c*__*l*__ represents the regression coefficients of *CN*_*g*_, *DM*_*g*_, *TF*_*t*_, *miR*_*m*_, and *lncR*_*l*_, respectively. For the genes in the sample, only the differential expression in the tumor sample and normal sample (R SAM package fold-change [>2, < 1/2] FDR < 0.05) was used to train the linear model. We used a two-layer neural network to train each model and obtained the factor of a variable in each model. Only the TFs with a weight greater than a specific threshold were determined to construct our network.

By filtering the TFs of each differential list gene, we build a key candidate gene regulatory network among mutated and non-mutated samples. Further, by performing an XOR operation on these two networks, we obtained an irregular regulatory network of key candidate ones, which contained an abnormal regulatory gene group of key candidate genes. The XOR operation is defined as: edges in the network is related to a regulatory links; and links within only one network were retained.

In order to obtain the set of genes that were dysregulated by key candidate genes, we mapped genes in the irregular regulatory network and key candidate ones to a protein-protein interaction network, found genes of the irregular regulatory network of key candidate genes in a two-step wise of the PPIN, and used these genes as the abnormal regulatory gene group of key candidate genes. In this way, a total of 130 abnormal regulatory gene groups of key candidate ones were collected, of which 89 key candidate genes could be reached in our protein-protein interaction network. The corrected cumulative rank scores of the 89 key candidate genes were calculated in pairs, and a score matrix of the functional similarity of the gene groups was obtained.

### Clustering Algorithm Based on Corrected Cumulative Rank Score

**Figure 2:**
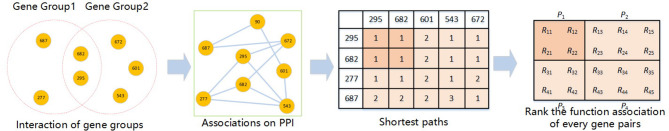
Corrected cumulative rank score calculation.

Clustering is often employed in various bioinformatics researches (61–68). The calculation process of the corrected cumulative rank score (CCRS) method (36) is shown in Figure 2. This method can measure the functional similarity between two gene groups by means of the functional relation and physical interaction between genes. This method defines the functional distance between two genes as the shortest path of two nodes in a PPIN. Then the distance of two genes reflects the similarity. Therefore, this method can be used to find the functional distance to score the functional similarity between two genes, and compare two gene groups by cumulatively scoring each pair of genes between the two gene groups. For any gene group, it must be completely consistent with its own functional similarity apparently. Therefore, when comparing two determined gene groups, there are two hypotheses: (1) any gene has a direct interaction to itself; (2) for two genes in the same gene group, if there is a path, the functional distance is independent of the path length. Based on the above two assumptions, when there is an intersection between two gene groups, the corrected cumulative rank score can thus be calculated.

We assume that there are gene groups *G*_1_ and *G*_2_. The intersection of *G*_1_ and *G*_2_ is defined as *G*. For the genes *p* and *q* taken separately from *G*_1_ and *G*_2_, there are *n* paths based on *m* nodes in the PPIN intersection *G*, and the corrected cumulative rank score is shown in formula (2).

(2) $$\begin{array}{l} CCRS\left(G_{1},G_{2}\right)=\frac{1}{N}(\sum_{\begin{array}{l} p\in G\\ q\in G \end{array}}R_{pq}+\sum_{\begin{array}{l} \;p\in G\\ q\in G_{1}-G \end{array}}R_{pq}+\sum_{\begin{array}{l} \;p\in G\\ q\in G_{2}-G \end{array}}R_{pq}\\ \;+\sum_{\begin{array}{l} p\in G_{1}-G\\ q\in G_{2}-G \end{array}}R_{pq}) \end{array}$$

*R*_*pq*_ is the functional distance from the gene *p* to the gene *q*, i.e., the shortest path length. *N* is the number of functional distances that exist. And *R*_*pq*_ = 0 if there is no path between gene *p* and *q*.

We used the corrected cumulative rank score as distance measure of the clustering algorithm, and then used the compactness clustering algorithm to find key gene modules. The distance measurement of the clustering algorithm at this time was as follows.

(3) $$\begin{array}{l} {\text{Dis}}_{ij}=CCRS\left(DS_{i},DS_{j}\right), \end{array}$$

Where Dis_*ij*_ is the distance from the *i*-th key gene to the *j*-th one, and *DS*_*i*_ and *DS*_*j*_, respectively, indicate the abnormal regulatory gene group affected by the *i*-th gene and the *j*-th gene. See Algorithm 1 for the clustering algorithm of key gene modules based on compactness.

**Algorithm 1:** Compactness-based module clustering algorithm

Input: dataset for n genes *G*; radius ε; neighborhood compactness threshold *m*
Output: gene modules with clustering lables
1. initialize *n* × *n* matrix *D*;
2. save key gene distances in *G* to *D*;
3. tag all genes as *core point* within ε radius higher than *m* in *G*;
4. tag all genes as *border point* around *core point* within ε radius;
5. tag all other genes as *noise point*;
6. foreach *core point* gene *p* which is not *visited*:
7. initialize a stack *s*;
9. set *p* as *visited*, then pushed into *s*;
10. while *s* is not empty:
11. *v* ← pop *s*;
12. foreach non-*visited* gene *q* **in** ε radius of *v*:
14. set *q* as *p* cluster;
15. set *q* as *visited*, then pushed into *s*;
17 end foreach
18. end while
19. end foreach
20. foreach *border point* gene *b*;
21. put *q* into any cluster of *core point* within ε radius;
22. end foreach

This algorithm is a clustering algorithm for the connection area, it defined cluster as the largest set of the compactness of genes connected, which divide cluster with sufficiently high compactness region. The basic idea is to optionally choose a gene *x*, the number of genes within ε radius if *x* region contains greater than a threshold and create a new cluster. This algorithm finds key gene modules for the key genes proposed in this paper and the abnormal gene groups regulated by it. The two parameters of the scanning radius and the neighborhood compactness threshold in algorithm 1 is automatically determined by methods in (69).

## Results

### Key Gene Modules

**Figure 3:**
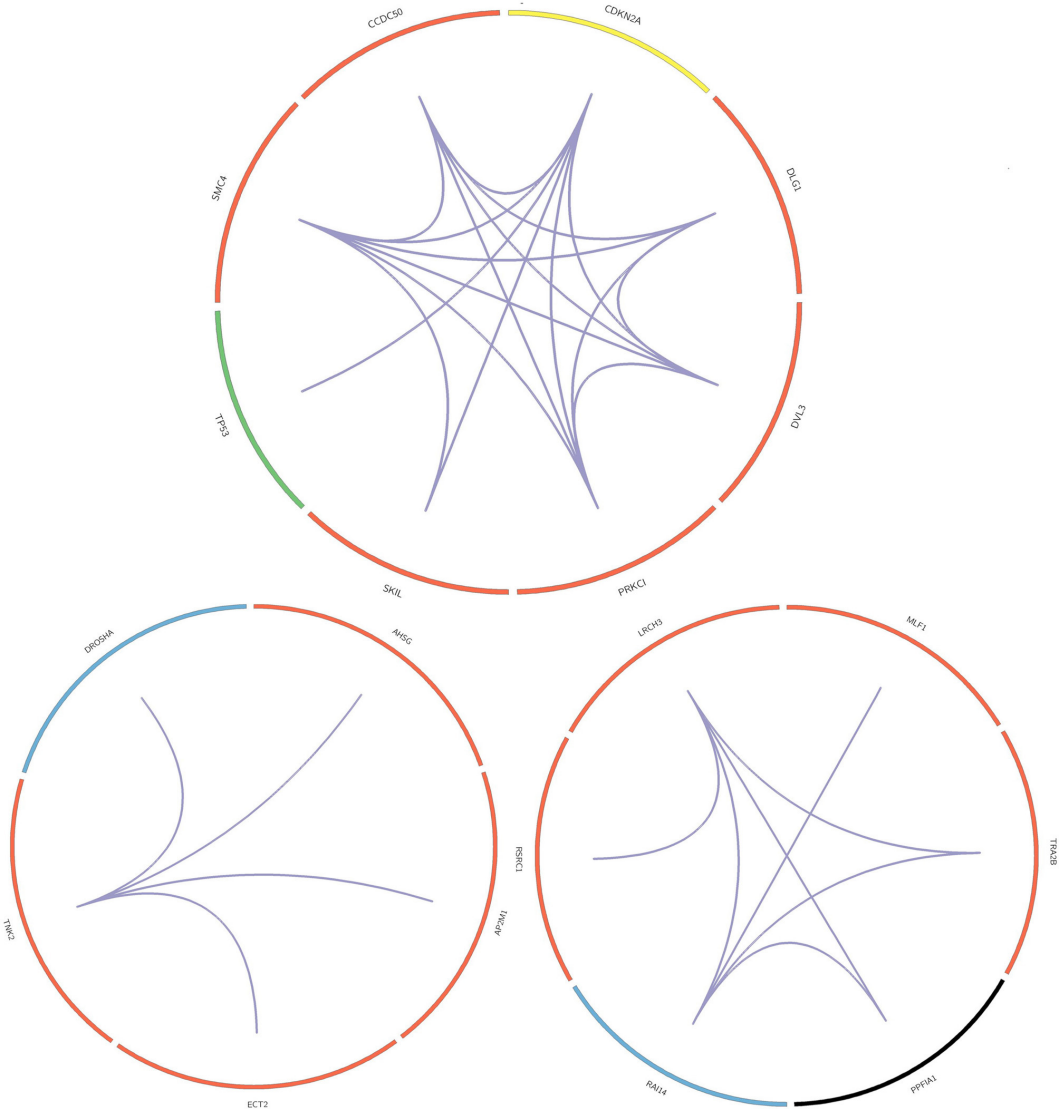
Core gene module.

In this paper, we mined key gene modules on an abnormal regulatory gene group of 89 key genes. In this way, we conclude three key gene modules with 19 genes, including *CDKN2A, DLG1, DVL3, PRKCI, SKIL, TP53, SMC4*, and *CCDC50; DROSHA, AHSG, AP2M1, ECT2*, and *TNK; LRCH3, MLF1, TRA2B, PPFIA1, RAI14*, and *RSRC1*. Among them, the 4 genes *PPFIA1, DROSHA, CDKN2A*, and *TP53* were related to LUSC (downloaded from the LUSC gene list from Cosmic, OMIM, HuGE, and GAD). There were 12 genes related to LUSC in GeneCards, as shown in Table 3. The relationship between the genes in the key gene module and the chromosomes they belong to are shown in Figure 3.

**Table 3:** Relevance of core genes to LUSC.

**Gene**	**Description**	**Gifts**	**Relevance**
TP53	Tumor Protein P53	79	160.57
CDKN2A	Cyclin Dependent Kinase Inhibitor 2A	71	129.25
PRKCI	Protein Kinase C Iota	74	21.52
ECT2	Epithelial Cell Transforming 2	60	7.91
TNK2	Tyrosine Kinase Non-Receptor 2	65	5.68
AHSG	Alpha 2-HS Glycoprotein	61	4.23
MLF1	Myeloid Leukemia Factor 1	60	4.17
DLG1	Discs Large MAGUK Scaffold Protein 1	62	4.16
DROSHA	Drosha Ribonuclease III	57	4.03
PPFIA1	PTPRF Interacting Protein Alpha 1	52	3.37
AP2M1	Adaptor Related Protein Complex 2 Mu 1 Subunit	63	3.27
SKIL	SKI Like Proto-Oncogene	64	2.89

The three key gene modules in Figure 3 have a total of 19 key genes. Of these, 18 were associated with lung cancer and 9 were associated with lung squamous cell carcinoma. The bar entities in the outer circle represents key genes. Among these, red, blue, yellow, black, and green represent the genes belonging to chromosomes 3, 5, 9, 11, and 17, respectively, and purple lines represent genes and the relationship between genes.

Most of the genes in these three modules are related to lung cancer, and there were key genes related to lung squamous cell carcinoma. A small number of genes have not been yet confirmed to be related to lung cancer, but they were all related to complex diseases or cancer. By investigating the module information to which these genes belong on NCBI, we found that most of the genes could be found on chromosome 3, and the specific information is shown in Table 4.

**Table 4:** Chromosome information of key genes.

**Gene name**	**Chromosome**	**Start site**	**End site**
TP53	hs17	7668402	7687550
CDKN2A	hs9	21967752	21995043
DROSHA	hs5	31400494	31532175
RAI14	hs5	34656328	34832612
PPFIA1	hs11	70270687	70384501
RSRC1	hs3	158110052	158544835
MLF1	hs3	158571162	158606460
SMC4	hs3	160399304	160434962
PRKCI	hs3	170222432	170305982
SKIL	hs3	170357678	170396849
ECT2	hs3	172750682	172829273
DVL3	hs3	184155311	184173614
AP2M1	hs3	184174846	184184091
TRA2B	hs3	185914568	185938136
AHSG	hs3	186612928	186621318
CCDC50	hs3	191329082	191398670
TNK2	hs3	195863364	195909009
DLG1	hs3	197042560	197299272
LRCH3	hs3	197790855	197889346

### Distinguishing Samples From Normal and Tumor

**Figure 4:**
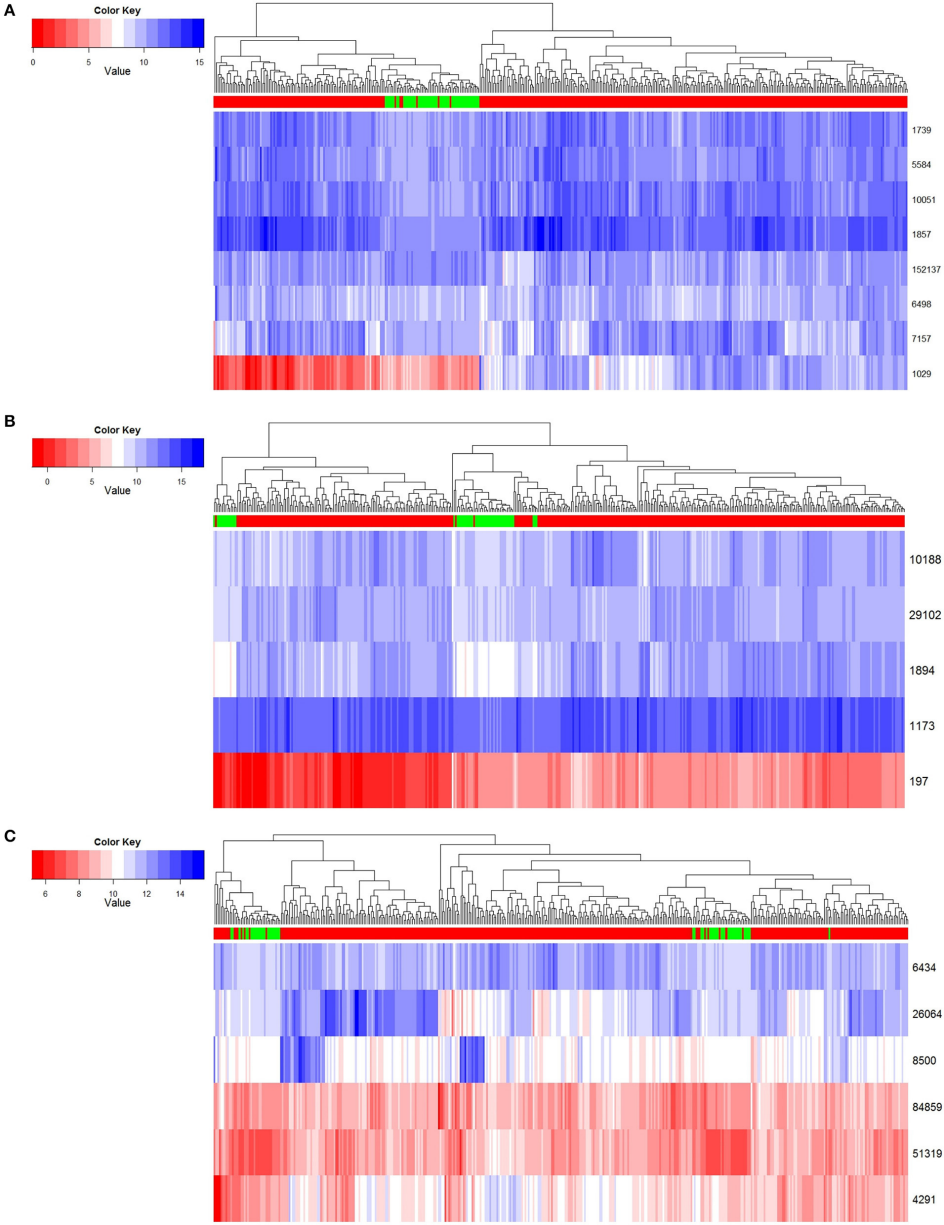
Normal and tumor samples resolved based on core genes. **(A)** Module 1 clustering result. **(B)** Module 2 clustering result. **(C)** Module 3 clustering result.

In order to exhibit the character of the three key modules we found in the development progress of LUSC, we hierarchically clustered the gene expression profile data from a total of 389 TCGA-LUSC samples, which included 337 tumor samples of TCGA-LUSC and 52 normal ones. For the distance measure, Euclidean distance was used to calculate the average distance between classes. The clustering analysis shows in Figure 4.

In the first row of Figure 4, red indicates that the sample is a tumor sample, and green is a normal one. Genes are illustrated in rows of the remaining part, and expression levels are in columns by scale of colors in blue and red. According to the results shown in Figure 4, we found that these three key gene modules could distinguish cancer samples from normal samples very robustly. Among these key genes, module 1 had the best classification effect, followed by Module 2 and Module 3.

### Function and Pathway

For the key gene modules obtained by this method, we used DAVID software to perform KEGG pathway analysis and Gene Ontology function analysis. Among these classifications, only the Biological Progress was selected for functional analysis. The specific functional terms and pathway ID are list in Table 5, the prefix of GO is the function ID from Gene Ontology, and the prefix of each has the pathway ID from KEGG. The results shows that important carcinogenic effect. Among these, GO: 0071158, GO: 0045893, and GO: 0007050 disorders will aggravate the abnormal proliferation of cells and have extremely important effects. p53 is a tumor suppressor protein, and hsa04115 is a p53 signaling pathway closely related to cancer. Many genes in the Hippo signaling pathway represented by hsa04390 are tumor suppressor genes. hsa05203, hsa05212, hsa05214, hsa05219, and hsa05223 are relate with a viral carcinogenic mechanism, pancreatic cancer, glioma, bladder cancer, and non-small cell lung cancer, respectively. HTLV-I virus infection related with hsa05166 which associated with several cancers. hsa05200 itself represents a carcinogenic pathway. These show that the pathways and functions regulated by this gene module are also firmly associated to the development of LUSC.

**Table 5:** Functions and pathways.

**Id**	**Term**
GO:0007050	Cell cycle arrest
GO:0045197	Establishment or maintenance of epithelial cell apical/basal polarity
GO:0045893	Positive regulation of transcription DNA-templated
GO:0046677	Response to antibiotic
GO:0070830	Bicellular tight junction assembly
GO:0071158	Positive regulation of cell cycle arrest
GO:0090004	Positive regulation of establishment of protein localization to plasma membrane
GO:0090399	Replicative senescence
hsa04115	p53 signaling pathway
hsa04390	Hippo signaling pathway
hsa05166	HTLV-I infection
hsa05200	Pathways in cancer
hsa05203	Viral carcinogenesis
hsa05212	Pancreatic cancer
hsa05214	Glioma
hsa05217	Basal cell carcinoma
hsa05219	Bladder cancer
hsa05223	Non-small cell lung cancer
GO:0070830	Bicellular tight junction assembly

### Survival Analysis

**Figure 5:**
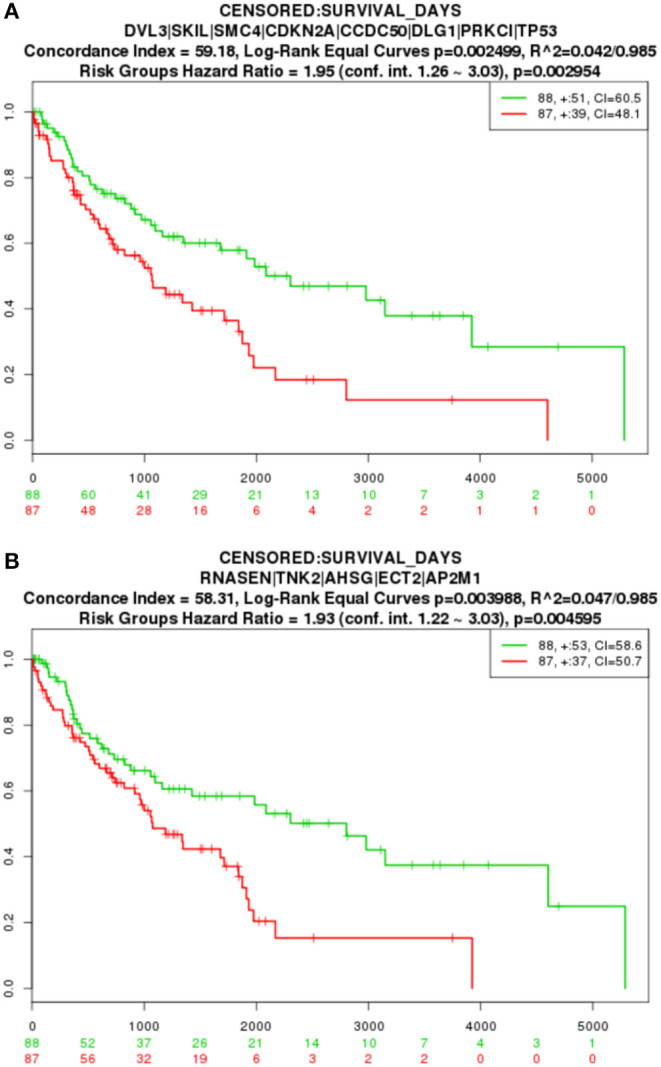
TCGA survival analysis result. **(A)** Module 1 survival analysis result. **(B)** Module 2 survival analysis result.

The key gene modules mined in this paper also have an important impact on LUSC and can characterize different risk level groups cancer samples. We applied a Cox proportional-hazards model to the genetic modification module for survival analysis. The Kaplan-Meier estimator curve is shown in Figure 5, where the abscissa represents days, and the ordinate shows the global survival rate. High- and low-risk group are in red and green line, respectively. The numbers in groups are shown in the legend and the loss to follow-up is indicated by “+” symbol. By comparing the two curves in the figure, we found that with increasing time, the two curves gradually split away, and the difference in survival time of the two groups of patients gradually increased, indicating that the 8 genes in module 1 (*p* = 0.002954) and the 4 genes in module 2 (*p* = 0.004595) can significantly distinguish the two groups of patients, are were highly relevant to the patient survival.

## Conclusions

In this paper, the corrected cumulative rank score method was used as a distance measurement for a compactness clustering method to mine the gene modules contained in key genes of abnormal regulatory gene groups. The corrected cumulative rank score helped us understand the interaction level of gene groups in a protein interaction network and calculate the functional similarity score between gene groups. Three key gene modules were mined in this paper, and more than half of the genes in each module were related to lung squamous cell carcinoma. In particular, in the first key gene module, the correlation between *TP53* as well as *CDKN2A* and lung squamous cell carcinoma ranks the top two on the GeneCards website, which fully illustrates the correlation between this module and lung cancer. The functions and pathways of these three modules have an important impact on the cause and progress of cancer. With survival analysis, we concluded that there were two key gene modules that could discriminate different risk level of patients very well. Our experimental results further validate the effectiveness of our overall method, and demonstrate the feasibility of mining key gene modules contained in a gene group using multiple methods.

## Data Availability Statement

The original contributions presented in the study are included in the article/supplementary material, further inquiries can be directed to the corresponding authors.

## Author's Note

The results in this article are based upon data generated by the TCGA Research Network: https://www.cancer.gov/tcga.

## Author Contributions

CW and KS conceived of the presented idea. CW and NZ drafted the manuscript. NZ and YZ designed the model and the computational framework and analyzed the data. KS supervised the project. All authors discussed the results and contributed to the final manuscript.

## Conflict of Interest

The authors declare that the research was conducted in the absence of any commercial or financial relationships that could be construed as a potential conflict of interest.
